# An Automated Three-Dimensional Detection and Segmentation Method for Touching Cells by Integrating Concave Points Clustering and Random Walker Algorithm

**DOI:** 10.1371/journal.pone.0104437

**Published:** 2014-08-11

**Authors:** Yong He, Yunlong Meng, Hui Gong, Shangbin Chen, Bin Zhang, Wenxiang Ding, Qingming Luo, Anan Li

**Affiliations:** 1 Britton Chance Center for Biomedical Photonics, Huazhong University of Science and Technology-Wuhan National Laboratory for Optoelectronics, Wuhan, Hubei, China; 2 MoE Key Laboratory for Biomedical Photonics, Department of Biomedical Engineering, Huazhong University of Science and Technology, Wuhan, Hubei, China; University of Maryland, College Park, United States of America

## Abstract

Characterizing cytoarchitecture is crucial for understanding brain functions and neural diseases. In neuroanatomy, it is an important task to accurately extract cell populations' centroids and contours. Recent advances have permitted imaging at single cell resolution for an entire mouse brain using the Nissl staining method. However, it is difficult to precisely segment numerous cells, especially those cells touching each other. As presented herein, we have developed an automated three-dimensional detection and segmentation method applied to the Nissl staining data, with the following two key steps: 1) concave points clustering to determine the seed points of touching cells; and 2) random walker segmentation to obtain cell contours. Also, we have evaluated the performance of our proposed method with several mouse brain datasets, which were captured with the micro-optical sectioning tomography imaging system, and the datasets include closely touching cells. Comparing with traditional detection and segmentation methods, our approach shows promising detection accuracy and high robustness.

## Introduction

Quantitative characterizations of the cytoarchitecture, such as cell size, location, density and spatial distribution, are fundamentally important for understanding brain functions and neural diseases. Rapid advances in optical imaging techniques have enabled scientists to visualize individual cells in massive image data of an entire mouse brain [1]. However, it is just impractical to manually count and locate all cells in the three-dimensional (3D) dataset of the entire mouse brain. An automated and accurate method is urgently needed to detect the centroid of each cell and obtain its contour [2].

Some automatic cell detection and segmentation methods in two-dimensional (2D) space have been proposed. However, the increasingly informative but complex 3D datasets have challenged the existing 2D approaches [3]. First, the brightness between adjacent 2D imaging sections is heterogeneous, which makes precisely extracting the foreground voxels very difficult. Second, cell morphology is varied and irregularly shaped, and some cells may closely touch.

There are already various image segmentation methods, and among them, threshold segmentation is the most common type. For example, the fuzzy threshold method [4] which relies on fuzzy sets is often used for image segmentation and can yield a stable threshold. However, the brightness between touching cells is very similar and obtaining their respective contours by this threshold is difficult. Thus, this method is not suitable for segmenting touching cells. Recently, super-pixel methods [5] have been proposed for image segmentation: a series of pixels with adjacent positions, similar color, brightness and other characteristics are used to compose a small area, and then this small area is further utilized for segmentation. Because touching cells have similar brightness and adjacent positions, using these small areas to segment them is difficult.

To solve the problem of cell touching in 3D images, a number of algorithms have been investigated. The early work in this field focused on watershed approaches. Although the traditional watershed algorithm can segment touching cells, it may lead to over-segmentation. The marker controlled and tensor voting watershed algorithms [6]–[8] have been proposed to overcome such limitations. Among these algorithms, the “markers” or “seed points” determined by a detection algorithm are a set of points in the image, usually one point per cell and close to the cell's center. These points are used by subsequent segmentation algorithms to delineate the spatial contour of each cell [3]. Indeed, the accuracy of the cell segmentation results depends on the reliability of the initial seed points. Several specialized seed point detection approaches have been proposed, including the famous iterative voting approach which relies highly on edge extraction, a gradient threshold and careful manual setting of parameters [3], [9]–[11]. The gradient threshold may be affected by heterogeneous brightness, resulting in over-segmentation. Moreover, the edge of a 3D image is very complex, and the direction of the radial gradient is irregular. Besides watershed and seed point detection techniques, level set (one of the deformed models) is also a traditional cell segmentation algorithm, and a modified coupled level set method has been proposed to segment touching cells [11]–[13]. However, coupled level set needs a suitable initialization contour to locate each touching object, and is difficult to extend to 3D images for a manually initialization surface is needed to locate each touching object. Gradient flow tracking, another extension of the deformed model method, has been proposed to segment touching cells. However, it is sensitive to heterogeneous brightness [3], [14]–[16], which may lead to inaccurate flow values and error direction, especially inside the cell where the gradient may not flow toward the cell center. More recently, researchers have introduced multi-scale LoG filtering which achieved good results for DAPI-stained slices [3]. As a modified version of multi-scale LoG, multi-scale cubic filtering has been employed to find sparse cells in fluorescent images [17], based on multi-scale filtering and extracting local maxima of the filtered image as a candidate cell centroid. Multi-scale filtering has achieved good detection results in fluorescent data, but the applicability of this method depends greatly on the signal to noise ratio [17]. All the aforementioned methods are based on gray scale or gradient information. To utilize the geometric characteristics of touching cells, a family of concavity detection algorithms can be applied to touching cells. Concave point detection is robust for detaching touching cells in a plane using one line to link two concave points [18]–[22]. However, it is difficult to directly extend to a 3D space because the splitting plane cannot be obtained based only on the detected points, as concave points often are not in the same plane.

To address the heterogeneous brightness distribution and closely touching cells in micro-optical sectioning tomography (MOST) datasets [23], this study presents a cell detection and segmentation method based on 3D seed points and a random walker algorithm. Our method first performs image preprocessing, which consists of image enhancement, binarization, noise elimination, morphological filtering and image filling. Then, the seed points are detected on a binary image. For touching cells, we detect the concave points and cluster these points, and a concave point clustering centroid (CPCC) based method is proposed to produce the seed points for touching cells. For isolated cells, a differential method is used to detect the seed points. Finally, a random walker algorithm, based on the seed points previously determined, is implemented to segment the cells, including the cells that touch. Our method has shown good performance both for clustered and isolated cells in the MOST datasets.

## Materials and Methods

### Nissl staining images of mouse brain

Three datasets of mouse brain images have been employed in this study, one from a Kunming mouse and two from C57BL/6 mice. The image datasets of entire mouse brain, Nissl stained [24], were captured by the MOST system. In this work, all animal experiments were approved by the Institutional Animal Ethics Committee of Huazhong University of Science and Technology.

The original voxel size was 0.35×0.4×1 µm, and the raw data of one entire mouse brain was greater than 2 terabytes. The raw data was processed by noise removal and brightness correction between adjoining slices to improve image quality [25]. Then, the voxel sizes of the Kunming and C57BL/6 datasets were resized to 0.5×0.5×0.5 µm and 0.35×0.35×0.35 µm, respectively, with cubic interpolation.

### Image Preprocessing

Because of the heterogeneous illumination and staining, some preprocessing approaches were adopted to enhance the image contrast and make the following binarization step perform better. The image contrast was enhanced in a linear manner: supposing r_min_ and r_max_ denoted the minimum and maximum intensity level in the original image, a transformation function stretched the levels linearly from original range (r_min_, r_max_) to the full range (0, L-1) [26] and L is 256 for an 8-bit image, and the transformation function is *imadjust* in MATLAB [27]. To automatically separate the foreground targets (cells) from the background voxels, we used an adaptive threshold method called Otsu binarization (using the *im2bw* function in MATLAB) [28] which required no additional parameters.

As a result of the heterogeneous image contrast, some background noise may be regarded as cell targets in the binary image. Thus, connected-component was extracted (using the *bwlabeln* and *regionprops* functions in MATLAB) by labeling the adjacent voxels and these voxels formed a connected-component [26]. Those connected-components for which the volume is too small was considered as noise and eliminated. Due to the heterogeneous brightness distribution, some cells in the binary image would contain holes. Therefore, morphological opening operation and hole filling were implemented [26], using the *imclose* and *imfill* functions in MATLAB.

After preprocessing, the following seed point detection and image segmentation steps were performed on the preprocessed binary image.

### Seed Point Detection

**Figure 1:**
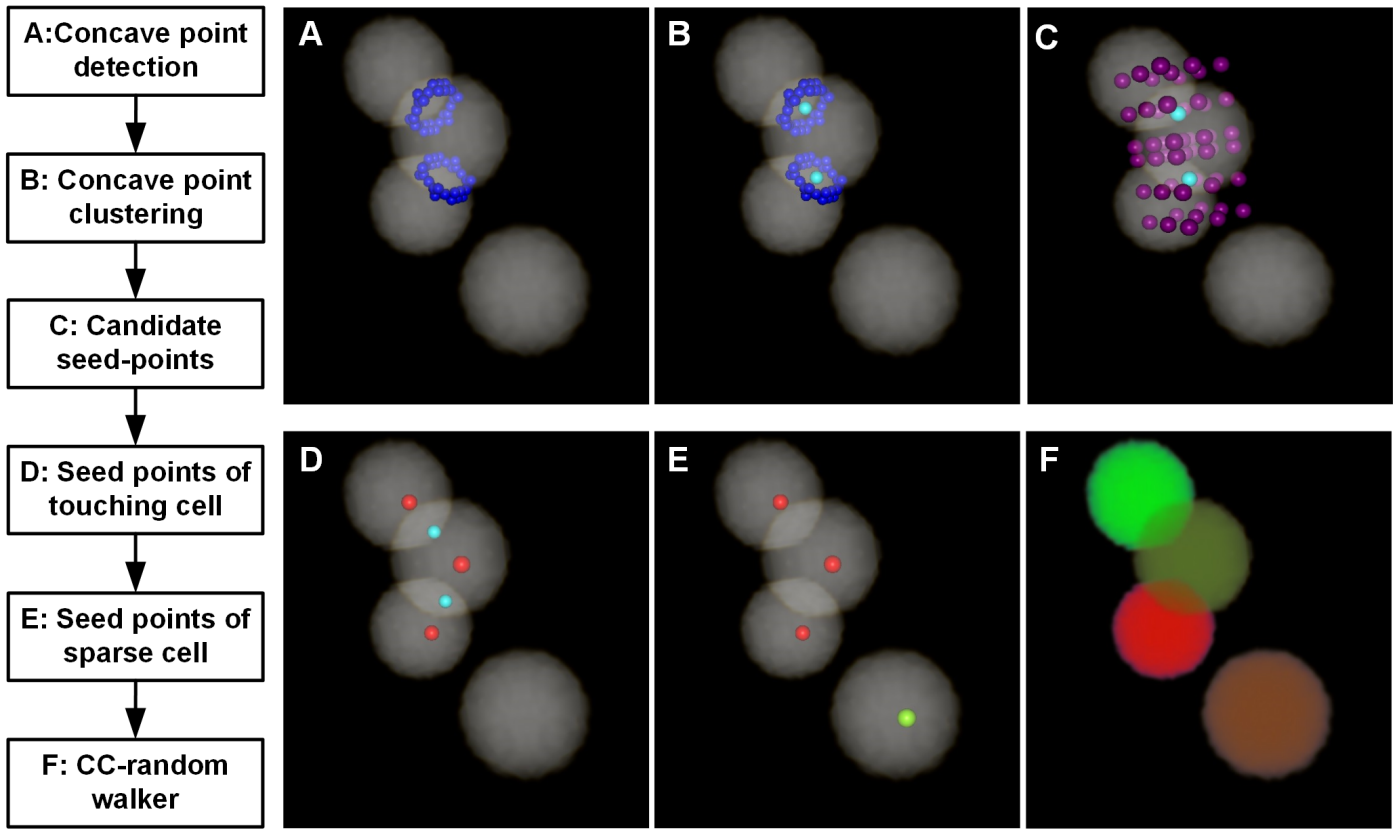
Workflow of seed point detection and segmentation in a 3D synthesized binary image stack. A–E are volume rendered with the color-map's alpha values of 0.2. (A) The concave point detection (blue points) results of the binary image. (B) The concave point clustering results. All the concave points are categorized into two classes: the points on the junction of the top two touching cells are categorized into the *L_1_* class and the other points on the junction of the bottom two touching cells are categorized into another class, *L_2_*. The light green color points are the clustering center of each class of concave points, *Ĉ_1_* and *Ĉ_2_*. (C) The 26 cubic neighbor points (reddish purple points) of each class of CPCC points. (D) The seed points of touching cells (red points), chosen from (C) with some restricted conditions. (E) The seed points of isolated cells (the light yellow points) are the local maxima points of the Gaussian-convoluted image. (F) Results of the CC-random walker segmentation, with different cells labeled as unique random colors.

The seed points were detected based on the binary image, and the outlined workflow is in Figure 1. It primarily includes touching cell and isolated cell detection. We have proposed a CPCC method to obtain touching cells' seed points, and we extracted the local maximum points of the Gaussian-convolved image as isolated cells' seed points. The CPCC method includes four primary steps: 1) detection of concave points, 2) clustering of concave points, 3) generation of candidate seed points, and 4) identification of the seed points of touching cells. To clearly describe the seed-point detection method for touching cells, a 3D synthesized binary image stack was used to show the processing results of each step above in Figure 1. All the image stacks in this study were volume-rendered with the AMIRA software [29].

#### Concave points detection

Han defines “concave points” as the discontinuous points on the boundaries of two overlapping objects [30]. Then, Bai et al used this definition to express the points on the junction boundaries of two touching cells [31]. In this study, the concave point detection algorithm was modified from a previous method [18]. The main principle of determining concave points is to calculate concaveness values of all the contour points, and then threshold those concaveness values. Each point of the contour has a concaveness value calculated as:
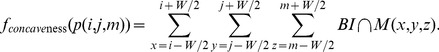
(1)


**Figure 2:**
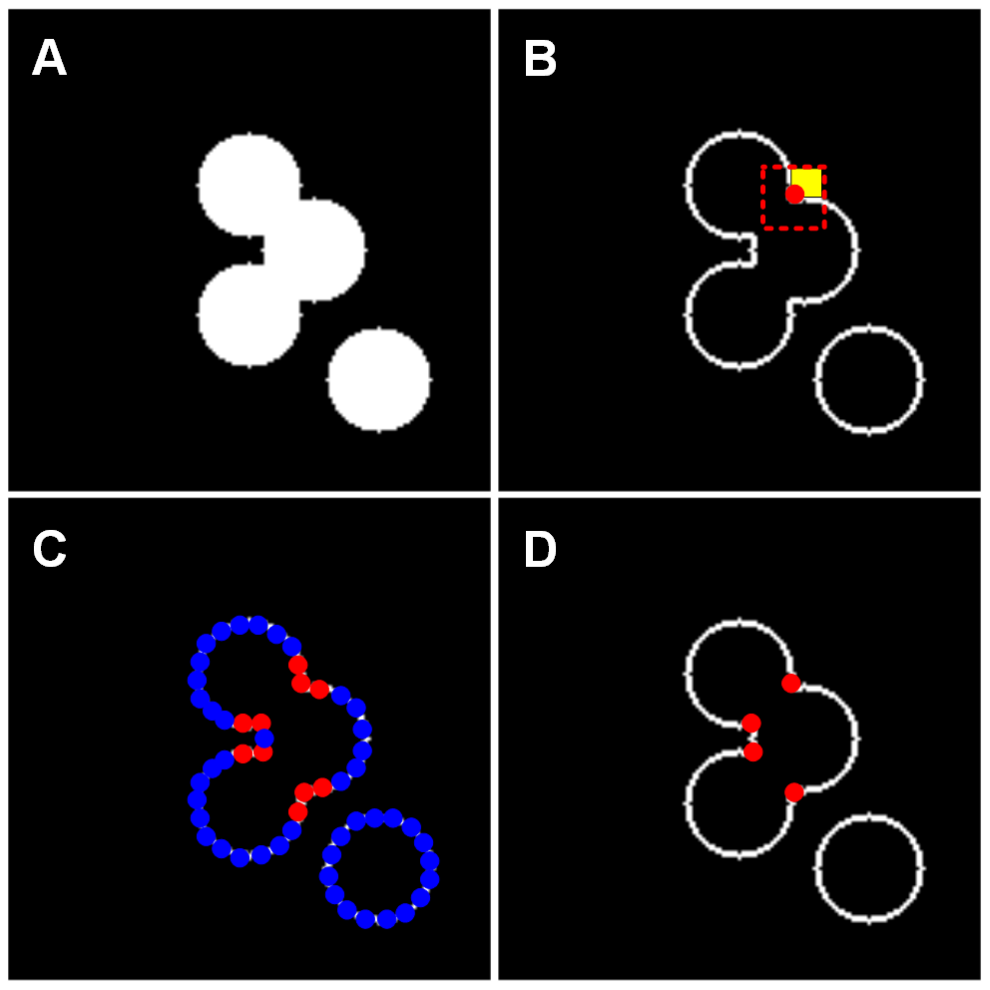
Schematic diagram of concave point detection in a 2D plane. (A) Binary image. (B) Contour image. The red point is on the contour, and the red dashed box is a mask centered at the red point. The yellow portion is where the mask intersects the background voxels of the binary image. (C) The candidate concave points (red points) meet the threshold condition. (D) The concave points (red points) meet the minimum concaveness value of its adjacent points.

Where *BI* is the binary image, *p* (*i, j, m*) is a point runs over the contour and *M* is a *W*×*W*×*W* (*W* = 5) mask centered on *p* (*i, j, m*) [18]. The concaveness value of a point, *f_concaveness_*(*p* (*i, j, m*)), is the number of voxels of *M* that intersects the background of *BI*. In Figure 2B, the red dashed box is the mask centered on red point *p* (*i, j, m*) and *f_concaveness_*(*p* (*i, j, m*)) is the yellow portion of the mask. We calculated the concaveness values of all the contour points (all the blue and red points in Figure 2C), then thresholded the concaveness values (*f*_concaveness_<*T* = 0.3, a little bigger threshold also works) of all the points [18] to produce the candidate concave points, i.e., the red points shown in Figure 2C. If N adjacent candidate points satisfy the threshold condition, the final concave point, *C* (individual red point shown in Figure 2D), is the point possessing the minimum concaveness value in its adjacent candidate points:
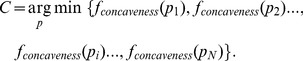
(2)By subtracting *f*_concaveness_(*p*) and adding the volume (*W^3^*) of *M*, then equation (2) can be converted to a maximization equation:
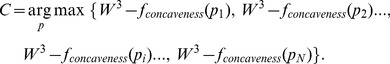
(3)In this way, the non-maxima suppression method can be used to obtain the local maxima point as the concave point.

We used 3D synthesized binary images to show these concave point detection results. If two cells were touching each other, we called them a touching-cell-pair. In Figure 1A, there are three touching cells (two touching-cell-pairs, i.e., the top touching-cell-pair and bottom touching-cell-pair) and the blue colored points are the detected concave points. For each touching-cell-pair, there were more than two concave points, but we could not directly connect any two of these concave points to segment the touching cells.

#### Concave points clustering

The objective of the concave point clustering proposed here is to cluster the concave points into corresponding classes. Concave points that connect the same touching-cell-pair are clustered in the same class; in Figure 1A, the concave points on the junction of the top touching-cell-pair should be clustered into one class, and the concave points on the junction of the bottom touching-cell-pair should be clustered into another class. Ideally, the concave points are located around the junction of the touching cells. Because of heterogeneous brightness, “false” concave points may appear on the surface of isolated cells. Thus, the clustering method should be able to cluster the concave points around the junction of the touching cells while avoiding “false” concave points. This study employed the DBSCAN algorithm [32]–[33] that has been widely applied in the data mining field. Due to the irregular shape and heterogeneous brightness of touching cells, the initial method would have led to incorrect results. For example, the concave points between two different touching cells might be clustered into the same class. This study has modified the DBSCAN algorithm in two aspects.

#### Constraint rule

The concave points clustered into the same class should be in the same connected-component of the binary image. In the previous step, suppose the number of detected concave points is D, i.e., *C_1_, C_2_…, C_i_…, C_j_…, C_D-1_, C_D_*, and we consider *C_Data_* as the set of the D concave points. Suppose that there are N connected components in the binary image, *T_1_, T_2_…, T_k_…, T_N-1_, T_N_*. If two concave points, *C_i_* and *C_j_*, are clustered into the same class, they must be located in the same connected component, i.e., to satisfy the constraint rule of *C_i_∈T_k_* & *C_j_∈T_k_*.

#### Kernel function

To overcome the irregular shape and heterogeneous brightness, a kernel function, *K*: *R* ^n^→[0, +∞), was introduced to DBSCAN algorithm and termed it Ckernel-DBSCAN. The kernel function is satisfied with three conditions: symmetry, locality and convergence. A Gaussian kernel function is selected in this study as:
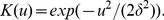
(4)


Where δ is the width parameter of the function and controls the radial scope (δ = 3), and *u* is the input variable. Some definitions of the DBSCAN algorithm are given [32]: (1) *Eps*, a distance threshold; (2) *S*_Eps_-neighbor of a point *C_i_*, denoted by *S_Eps_(C_i_)*, is defined by *S*_Eps_(*C_i_*) = {*C_j_*∈*C_Data_*|dist(*C_i_*, *C_j_*)<*Eps*}; (3) *Minpts*, a number threshold, is the minimum number (*Minpts*) of points that should be in *S_Eps_(C_i_)*.

After considering the constraint rule of *C_i_*∈*T_k_* & *C_j_*∈*T_k_* and introducing the kernel function and the definitions of the DBSCAN algorithm, the density of a concave point C*_i_* is defined as:
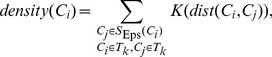
(5)where *C_i_* and *C_j_* are concave points. The constraint rule *C_i_∈T_k_* & *C_j_∈T_k_* indicates that *C_i_* and *C_j_* are in the same connected component.

From the modified definition of density, it is evident that those points, which are nearer to the point *C_i_*, have a greater impact on *density(C_i_)*, whereas other points, which are far away from point *C_i_*, have almost no impact on *density(C_i_)*. Additionally, only the points that locate in the same connected component with point *C_i_* have an impact on *density*(*C_i_*), which avoids the impact of false concave points on isolated cells.

Now, we redefine the term *density-reachable*. A point *C_i_* is *density-reachable* from a point *C_j_* if *C_i_∈S_Eps_(C_j_)* & *density(C_j_)*≥*Minpts*. The definitions of *noise points*, *core point* and *border point* in DBSCAN algorithm are given [32]: *noise points* are the points not belonging to any of these clusters; *core points* are the points inside of the cluster; and *border points* are points on the border of the cluster. There are two parameters in this algorithm, *Eps* and *Minpts*, that influence the clustering results; thus, we chose suitable values like the DBSCAN algorithm did (here: *Eps* = 3, and *Minpts* = 5). The Ckernel-DBSCAN algorithm is as follows:

**Ckernel-DBSCAN Algorithm**

1. {*C_Data_*} is the set of all the concave points;
2. Make *n* = 0;
3. *while* {*C_Data_*}≠0 do
4. Choose one concave point *C_i_*∈{*C_Data_*} and compute *density*(*C_i_*);
5. if *C_i_* is not a *core point*
6. Label *C_i_* as a *noise point* and delete *C_i_* from the set {*C_Data_*}: {*C_Data_*} = {*C_Data_*}−*C_i_*;
7. e*lse if C_i_* is *core point*
8. *n*: = *n*+1;
9. Find the concave point *C_j_*: (*C_i_∈T_k_*) & (*C_j_∈T_k_*) and *C_j_* is *density-reachable* from *C_i_*; Cluster *C_i_* and *C_j_* into class *L_n_*. Early *border points* that may be labeled as *noise points* are also clustered into class *L_n_*. Delete *C_i_* from the set:{*C_Data_*} = {*C_Data_*}−*C_i_*;
10. end {*if*}
11. end {*while*}

All the concave points are clustered using the Ckernel-DBSCAN algorithm and assume that the number of clustering classes is *n (1, 2…, m…, n)*. For the class *L_m_*, we calculate the centroid point, *Ĉ_m_* (referred to as the concave point cluster centroid, i.e., the CPCC), and there are *V* concave points belonging to class *L_m_*. Then, *Ĉ_m_* is calculated as:
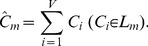
(6)


The concave points clustering results of the 3D synthesized binary image are shown in Figure 1B. The points on the junction of the top touching-cell-pair were categorized into class *L_1_*, and those of the bottom touching-cell-pair were categorized into class *L_2_*. The two light green colored points are the clustering centroids of the two classes, i.e., *Ĉ_1_* and *Ĉ_2_*.

#### Candidate seed points of touching cells

**Figure 3:**
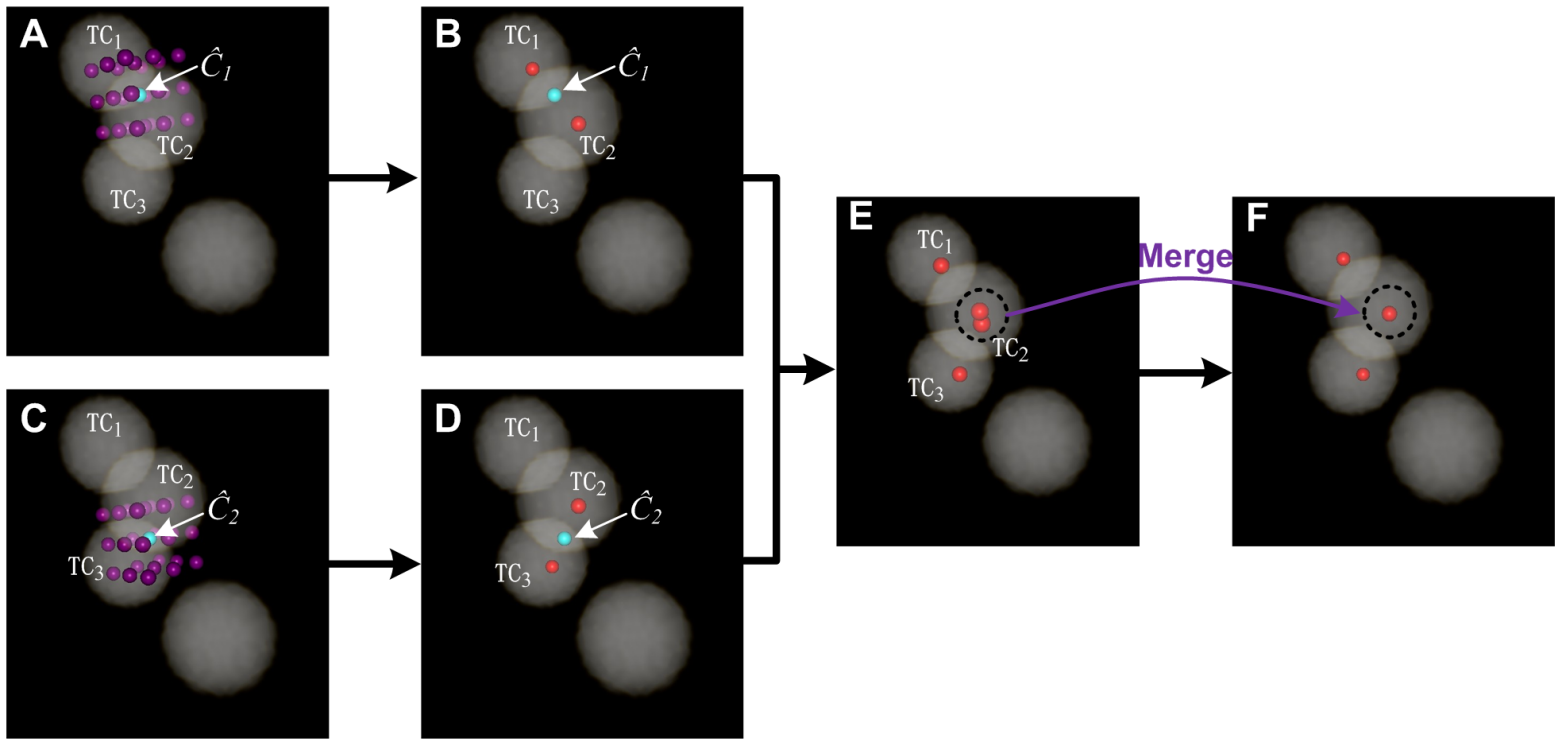
Seed points chosen from candidate points in a 3D synthesized binary image stack. The stack is volume-rendered with the color-map's alpha values of 0.2. The three touching cells are named TC_1_, TC_2_ and TC_3_, and the light green points, *Ĉ_1_* and *Ĉ_2_*, are the clustering centers of each class of concave points. (A) The reddish purple points are the 26 cubic neighbor points of the first CPCC point, *Ĉ_1_*. (B) The red points are the seed points chosen from the reddish purple points from (A) under some restricted conditions. (C) The reddish purple points are the 26 cubic neighbor points of the second CPCC point, *Ĉ_2_*. (D) The red points are seed points chosen from the reddish purple points of (C) under some restricted conditions. (E) The total seed points of (B) and (D). (F) The merge of all the seed points in (E) under some restricted conditions.

Next, we employed a strategy to obtain candidate seed points of touching cells. There is a method of determining them [34], but we used the CPCC point, *Ĉ_m_*, to construct a cube whose side-length is 2×*Rc* (*Rc* corresponds to the cell radius, *Rc* = 7 µm) and the cube is centered on *Ĉ_m_*. Then, total 26 key points of the cube were obtained, i.e., the eight vertexes, the centers of the six faces and the midpoints of the twelve sides. The 26 points are similar to the 26-connected neighbors of *Ĉ_m_*. We called them the 26 cubic neighbor points of *Ĉ_m_* and defined *S_D_* as the set of the 26 points: *S_i_∈S_D_ (0≤i≤25)*. Suppose the coordinates of *Ĉ_m_* are (*x, y, z*), the coordinates of *S_i_* are (*x_i_ = x*±*Rc, y_i_ = y*±*Rc, z_i_ = z*±*Rc*) and *S_i_* is the candidate seed point. In the 3D synthesized binary image stack, as shown in Figure 1C, the reddish purple points are the cubic neighbor points of two light green colored CPCC points, *Ĉ_1_* and *Ĉ_2_*, and each CPCC point has 26 neighbor points. We show *Ĉ_1_* and its 26 neighbor points alone in Figure 3A, and the three touching cells are named TC_1_, TC_2_ and TC_3_. The reddish purple points are the 26 cubic neighbor points (candidate seed points) of the first CPCC point, *Ĉ_1_*. In Figure 3C, the reddish purple points are the 26 cubic neighbor points (candidate seed points) of the second CPCC point, *Ĉ_2_*.

#### Seed points of touching cells

The final seed points were chosen from the 26 cubic neighbor points (*S_D_*) of *Ĉ_m_*. Ideally, one CPCC point only corresponds to two seed points, i.e., 24 neighbor points (candidate seed points) of *Ĉ_m_* are redundant. For example, only two of the 26 neighbor points of *Ĉ_1_* are needed in Figure 3A, with one corresponding to cell TC_1_ and the other one corresponding to cell TC_2_. For *Ĉ_m_*, the seed points were selected from *S_D_* using some restricted conditions. A candidate seed point, *S_i_∈S_D_*, with coordinates (*x_i_, y_i_, z_i_*), was a seed point only when it satisfied the following conditions: (1) *BI*(*x_i_, y_i_, z_i_*)>0, where *BI* is the binary image and the final seed point must be situated in the foreground; and (2) *A*(*x_i_, y_i_, z_i_*)*>σ = Rc*, where *A* is the Euclidean distance map of *BI* and we chose the candidate seed points nearest to the cell center. After the two conditions above, the last seed points, S*, of the CPCC point *Ĉ_m_* are as follows:
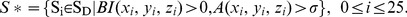
(7)The final condition, (3), is that the distance between any two seed points must be greater than the radius of a cell, *Rc*. Otherwise the two seed points will be merged into a single point.

In Figure 3B, two seed points (the red points) were achieved by choosing from the candidate seed points (reddish purple points) in Figure 3A under the restricted conditions (1–2). In Figure 3D, there were also two seed points achieved by choosing from the candidate seed points (reddish purple points) in Figure 3C. Both *Ĉ_1_* and *Ĉ_2_* produced one seed point for cell TC_2_ in Figure 3E, however, the distance of the two seed points was less than *Rc*, and the two points were merged into one seed point (the red point inside the cell TC_2_) in Figure 3F.

In this study, we used a differential method to detect isolated cells, which has no concave points in the connected component of the binary image; thus, seed point detection was performed on the binary images that have eliminated the connected components with concave points. First, we convolved the binary image with a Gaussian template (17×17×17, a larger size also works) and then extracted the local maxima of the convolved image, and these local maxima points were considered as seed points. The seed points of isolated cells of the 3D synthesized binary image stack were detected using the local maxima of the Gaussian-convolved image and are displayed in Figure 1E (the light yellow point).

### Connected Component-Based Random Walker Segmentation

Cell contour segmentation was based on the seed points obtained in the previous step and was implemented on the binary image. Grady has proposed a random walker segmentation method [35] which has been applied to medical image for interactive segmentation. Initial seed points must be set by the user, and then, the voxels are assigned to a given seed point according to a maximum probability. The memory usage of the random walker algorithm is very high and this algorithm requires to set seed point manually. The source code can be downloaded from www.cns.bu.edu/~lgrady/random_walker_matlab_code.zip. We have modified the original algorithm to make it more feasible and convenient in our situation, termed the modified algorithm of a connected component-based random walker (CC-random walker) segmentation method. Each time, only one connected component in the image, rather than the entire image, was processed. Thus, only the seed points in the current connected component would be considered. There are primary four steps in our CC-random walker cell segmentation method:

#### (1) Construct the edge weights network diagram

Grady applied the Gaussian function as the weight function. For the binary image in our dataset, the weight function can be simplified to:
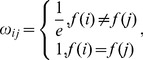
(8)where *i* is the binary image voxel, *f(i)* is the value of the voxel and *e* is the natural constant.

#### (2) Obtain a set of label points

Suppose N-1 seed points are generated in the seed point detection step, and we randomly catch one point in the background of the binary image. The N-1 seed points and the background point together compose the set of N label points, and all the remaining voxels in the binary image are un-labeled points.

#### (3) Solve the Laplacian matrix and the combinatorial Dirichlet problem

The combinatorial Laplacian matrix is defined in [35]:
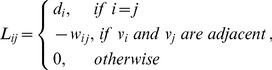
(9)and the Dirichlet problem is:
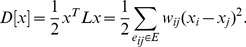
(10)


The detailed solution process for *D[x]* is described in [35] and its solution assigns each un-labeled point to a given label point according to a maximum probability value. This labeling means the un-labeled point and the corresponding label point belong to the same cell. As long as all the remaining un-labeled points are assigned, the cells are segmented.

#### (4) Process the connected component as a unit

Each time, we ran the random walker segmentation on only one connected component and iterated the previous steps (1–3) until all the remaining connected components were processed.

A sparse linear equation must be solved in step (3). This solving procedure requires a significant amount of memory. Although some methods have been proposed to optimize solving such sparse linear equations in the literature [36], [37], it is still very difficult to apply such methods to 3D image processing. At a time, the CC-random walker method only processes one connected component, instead of all the connected components. Consequently, the computational complexity may be increased.

The segmentation results of the 3D synthesized image data are shown in Figure 1F, where each cell is labeled with a unique random color. In particular, the colors of touching cells are different from each other.

## Results

To demonstrate the performance of our algorithm, we verified it with three datasets, one from Kunming and two from C57BL/6 mice brains. Six image stacks from the Kunming mouse (indexed from K1 to K6), and twenty image stacks from the two C57BL/6 mice (indexed from C1 to C20) were selected. All image stacks were selected from the barrel cortex of the mouse brain data, without overlapping each other. The sizes of K1 and K2 stacks were 67×68×15 µm and 100×100×15 µm, respectively. The sizes of K3–6 stacks were all 100×100×100 µm. For the two C57BL/6 mice, the sizes of C1–20 stacks were all 200×200×30 µm.

### Quantitative Validation of Cell Detection

The performance of the detection algorithm can be described in terms of precision and recall [17]. Manually marked cell centers were recognized as the ground truth. The detected cell was matched to a manually marked cell if the distance between their cell centers was below the cell radius, *Rc*. Then, the detected cell and the manually marked cell composed one unique pair (as one-to-one match). A cell is correctly detected if it one-to-one matches with a manually marked cell in the ground truth. Thus, we first matched each detected cell to manually marked cells and vice versa, assuming that there are *N_g_* cells of ground truth, about *N_d_* cells have been detected and *N_c_* of the *N_d_* cells made a one-to-one match with a manually marked cell. The definitions of recall and precision are as follows:
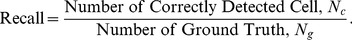
(11)

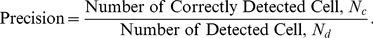
(12)


**Figure 4:**
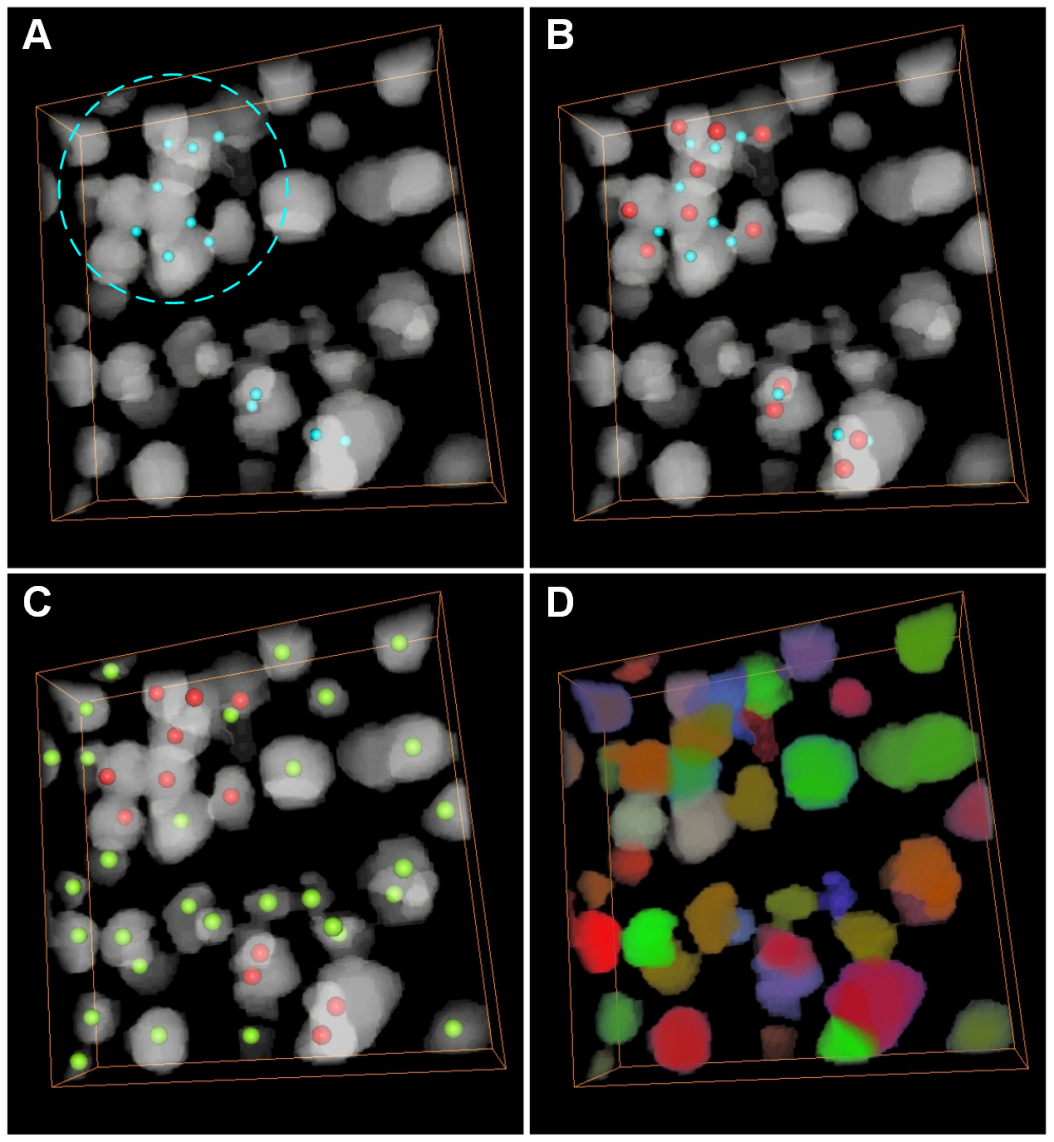
Cell detection and segmentation on the K1 stack. The stack is a preprocessed binary image and volume-rendered with the color-map's alpha values of 0.2. (A) The CPCC point result (light green points). The dashed circle indicates the cell-touching region. (B) The seed point (red points) of touching cells from the 26 cubic neighbor points of CPCC. (C) The seed points (light yellow points) of sparse cells. (D) Results of the CC-random walker segmentation, where different cells are labeled in unique random colors.

**Figure 5:**
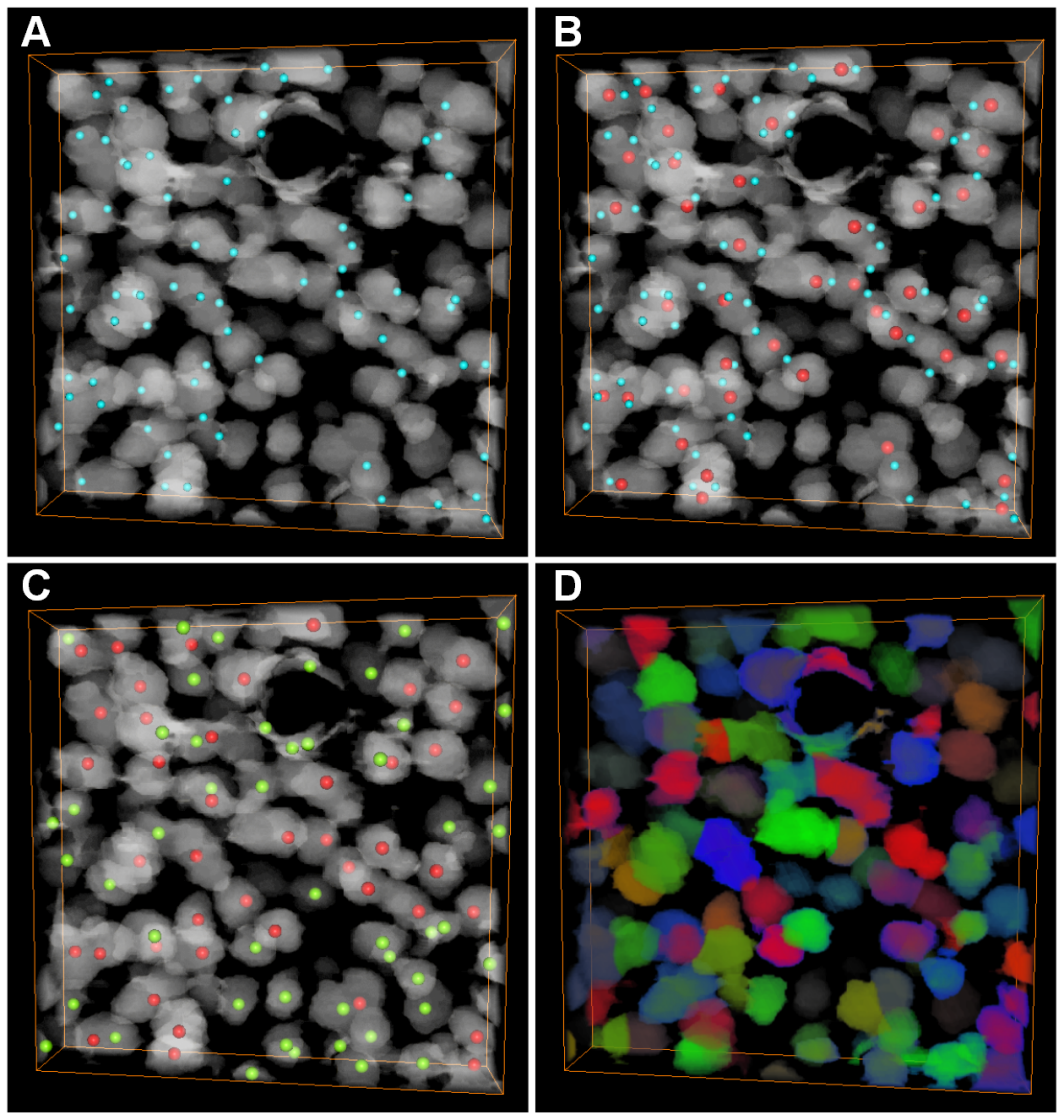
Seed point detection and segmentation on the K2 stack. The stack is a preprocessed binary image and is volume-rendered with the color-map's alpha values of 0.2. The black ring is a large vessel. (A) The results of the CPCC points (light green point). (B) The seed points (red points) of touching cells selected from the 26 cubic neighbor points of CPCC. (C) The seed points (light yellow points) of sparse cells obtained by extracting the local maximum of the Gaussian-convoluted image. (D) Results of the CC-random walker segmentation. Different cells are labeled in unique random colors.

The results of the proposed algorithm on the K1 stack are shown in Figure 4, and the CPCC points are displayed in Figure 4A. Twelve seed points (the red points in Figure 4B) of touching cells were detected based on the CPCC points, and thirty seed points of sparse cells were detected by local maximum of Gaussian-convolved image (the light yellow points in Figure 4C). With unique random colors, the cell segmentation results are shown in Figure 4D, and all cells are labeled. The results of the K2 stack are shown in Figure 5. Cells were mostly touching on the K2 stack. Fifty-four seed points (the red points in Figure 5B) were detected using the CPCC points, and fifty-six seed points of sparse cells were identified (the light yellow points in Figure 5C). The segmentation results of K2 are shown in Figure 5D.

In the following section, we compared our 3D cell detection algorithm with some 3D state-of-the-art methods, including the multi-scale LoG (MSL) [3], gradient flow tracking (GFT) [14] and TWANG [38] methods. The executable program of the GFT method has been described by Li *et al* [14] (http://www.cbi-tmhs.org/ZFIQ/download.htm). For the MSL method, we implemented it with the FARSIGHT Toolkit (http://www.farsight-toolkit.org/). The source code of the TWANG algorithm has been described by Stegmaier *et al* and can be downloaded as a supporting file (http://dx.doi.org/10.1371/journal.pone.0090036). The parameters of these algorithms are set to fit the datasets. We have presented only the values of the adjusted parameters, and the parameters with default values are not shown. For K1–K6 stacks, the main parameters of GFT method were *f* = 3, and for the C1–C20 stacks, the corresponding parameters were *f* = 2. For all the 26 stacks, the main parameters of MSL method were *σ_min_* = 5 and *σ_max_* = 8. For the K1–K6 stacks, the main parameters of the TWANG method were *σ_min_* = 5 and *σ_max_* = 8, and for the C1–C20 stacks, the corresponding parameters were *σ_min_* = 7 and *σ_max_* = 10. For all the 26 stacks, the main parameters of our proposed method were *Eps* = 3, *Minpts* = 5, and *Rc = 7* µm. The background interference in our dataset was substantial, and for a fair comparison, these algorithms were executed on the background-subtracted stacks. These background-subtracted stacks were the preprocessed binary image stacks with foreground voxels filled with original gray values.

**Figure 6:**
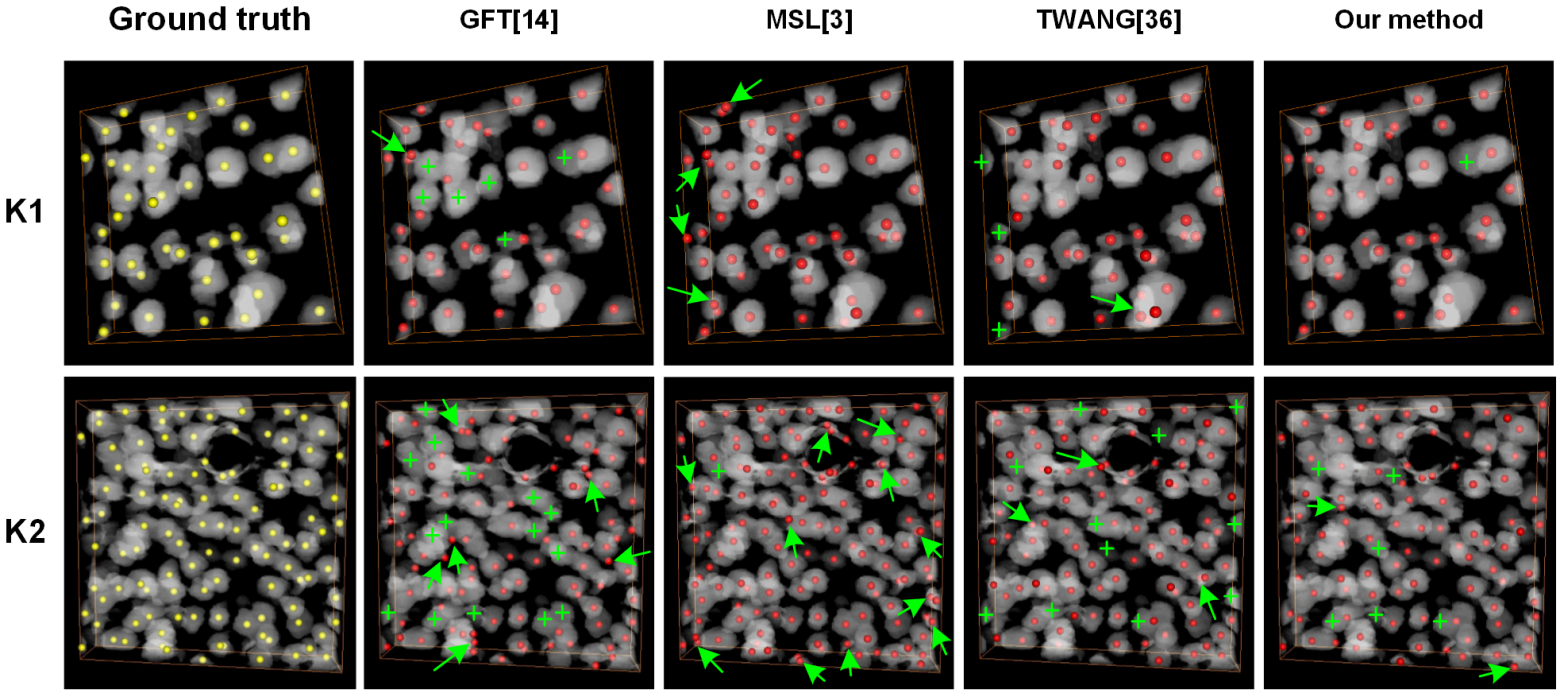
Comparison of different detection results achieved with the investigated methods on K1 and K2 stacks. The top and bottom rows represent the K1 and K2 stacks. The stacks are preprocessed binary images and are volume-rendered with the color-map's alpha values of 0.2. The yellow points are the ground truth, and the red points are cell centroids achieved through different segmentation methods. The green crosses indicated that the cells that are not detected. The green arrows indicated the false detected cells.

In Figure 6, we show the detected cell centroids produced by our method and the other three algorithms on the K1–K2 stacks. Manually marked cells were identified as the ground truth. The GFT method did not detect some cells, especially in cell-touching regions, and the green crosses label the undetected cells. The MSL method detected nearly all cells, even though some cells were closely touching. While this method detected redundant or false cells in both isolated and touching cell areas, false cells are marked with green arrows. The TWANG method also detected some touching cells but had both missing and false detected cells. Our proposed method had few missing and false detected cells. We will further analyze the comparison results in the discussion section.

In Table 1, we present the detailed statistics (precision, *P*, and recall, *R*) of the four quantitative detection results compared with the ground truth on the K1–K6 stacks. The average recall rate of the proposed method was 89.4±3.0%, and the average precision rate was 88.8±1.3%. However, for the GFT, MSL and TWANG methods, the average recall rates were 82.6±3.7%, 95.2±3.4%, and 86.1±4.2%, respectively, and the average precision rates were 82.6±3.7%, 79.4±3.7% and 85.0±6.2%, respectively. The average recall rate of the MSL method was the highest, and it could detect all the cells, but the precision rate was lowest because of the false detected cells, which are presented in Figure 6. The average recall rate of the GFT method was the lowest because of the undetected cells (Figure 6). For the TWANG method, both the recall and precision were lower than our proposed method, and our proposed method had the best trade-off between recall and precision.

**Table 1:** Performance of cell detection results using different methods on K1–6 stacks.

Image Stack	Number of Cell	GFT [14]		MSL [3]		TWANG [38]		Our method	
		*R*	*P*	*R*	*P*	*R*	*P*	*R*	*P*
K1	40	80.0	86.3	100	78.4	87.5	85.4	94.0	90.4
K2	98	80.6	84.0	96.0	80.3	82.7	85.1	87.8	88.4
K3	445	84.6	76.7	90.8	75.8	90.9	82.7	90.0	88.9
K4	387	82.7	80.0	95.6	81.4	87.1	74.7	89.1	89.9
K5	555	84.0	82.6	97.1	75.4	79.6	93.1	90.6	86.8
K6	507	70.8	85.7	91.7	85.1	88.9	89.0	85.0	88.2
Average	339	80.5±5.1	82.6±3.7	95.2±3.4	79.4±3.7	86.1±4.2	85.0±6.2	89.4±3.0	88.8±1.3

“*R*” means recall (%) and “*P*” means precision (%).

To further evaluate the proposed detection method, we employed our method on the C1–20 stacks. The detection results of all the 20 stacks are presented in Table 2. The overall average recall rate of the proposed method was 89.7±2.8%, and the average precision rate was 88.0±2.7%. For the GFT, MSL and TWANG methods, respectively, the average recall rates were 78.2±3.7%, 92.1±2.5% and 81.7±3.8%, and the average precision rates were 82.0±5.4%, 80.0±7.9%, and 83.5±6.1%. On these stacks, the multi-scale LoG method still produced the highest recall, the GFT method still had the lowest precision and our proposed method still had the best trade-off between recall and precision.

**Table 2:** Performance of cell detection results using different methods on C1–20 stacks.

Image Stack	Number of Cell	GFT [14]		MSL [3]		TWANG [38]		Our method	
		*R*	*P*	*R*	*P*	*R*	*P*	*R*	*P*
C1	506	81.8	81.7	93.9	81.5	84.6	84.2	91.1	86.6
C2	870	75.1	88.4	91.0	82.9	79.8	85.5	88.1	84.7
C3	419	84.2	75.7	93.1	70.0	79.7	79.2	91.6	88.3
C4	464	80.0	77.8	91.8	76.8	81.3	81.4	92.6	84.1
C5	721	80.0	85.3	94.6	84.9	84.9	89.1	92.2	89.1
C6	712	75.3	86.7	93.0	84.1	81.3	86.7	92.9	88.1
C7	496	81.5	74.0	89.7	71.8	72.6	80.8	89.5	88.1
C8	595	74.8	84.2	95.1	78.9	84.9	86.9	91.9	88.2
C9	859	71.0	85.3	90.0	88.0	77.6	88.9	89.4	87.1
C10	527	80.1	85.3	89.0	87.2	81.6	90.3	90.3	88.3
C11	892	72.0	90.5	90.9	92.3	79.3	94.3	86.0	94.2
C12	561	75.8	88.2	89.6	90.5	82.7	91.0	87.2	94.0
C13	749	74.1	85.2	91.3	85.7	79.6	88.8	83.6	88.0
C14	441	80.5	84.7	93.4	85.7	85.0	83.0	90.7	90.9
C15	362	76.5	75.4	88.1	68.5	83.1	78.2	87.3	88.0
C16	425	83.5	73.2	94.4	76.4	84.0	77.0	92.9	86.2
C17	372	77.4	76.2	92.7	65.8	80.6	74.4	87.6	83.4
C18	428	80.4	75.2	88.6	81.7	75.7	78.8	85.0	87.5
C19	403	81.4	84.3	94.8	79.6	86.6	80.9	92.1	88.3
C20	454	79.5	82.2	97.1	67.2	89.0	71.0	92.5	87.1
Average	562	78.2±3.7	82.0±5.4	92.1±2.5	80.0±7.9	81.7±3.8	83.5±6.1	89.7±2.8	88.0±2.7

“*R*” means recall (%) and “*P*” means precision (%).

### Qualitative Comparison of Cell Contour Segmentation Methods

In this section, we compared our cell segmentation algorithm with some state-of-the-art methods. Two metrics, including over-segmentation and under-segmentation, were utilized to evaluate the segmentation results. The over-segmentation metric indicates that a cell has been separated into more than one object. The under-segmentation metric indicates that touching cells have not been appropriately divided or that a cell is not segmented.

**Figure 7:**
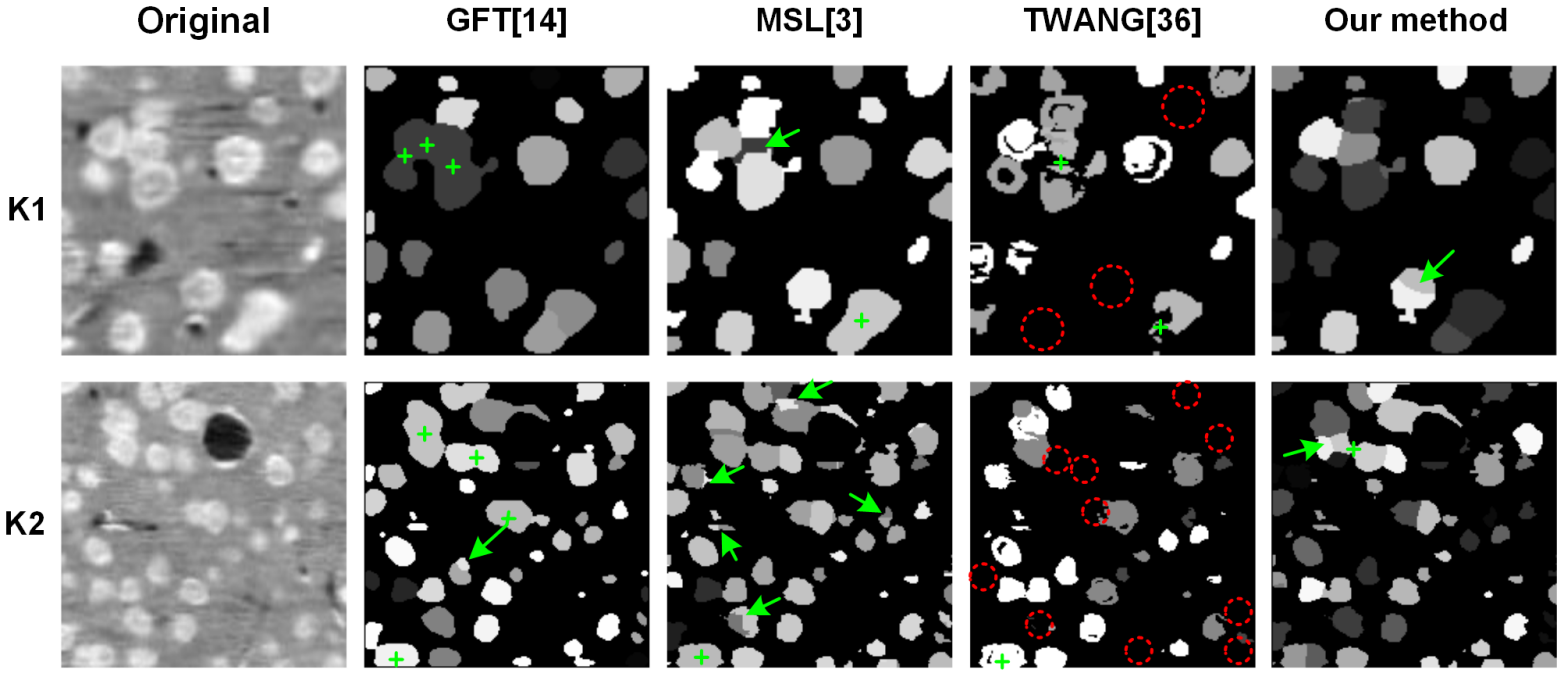
Comparison of different segmentation results achieved with the investigated methods on some two-dimensional slices. The slices are from the 3D original and segmented results of the K1 and K2 stacks. Each cell is labeled using one gray value in the segmentation results. The green crosses indicate the under-segmented cells. The green arrows indicate the over-segmented cells. The red dashed circles indicate that most of the voxels of one cell are not segmented.

In Figure 7, the performance of the proposed segmentation method was compared with the GFT, MSL and TWANG methods. The two representative slices were chosen from the K1 and K2 stacks and were adjusted to the same size. Each segmented cell is labeled with one gray value. The green crosses indicate under-segmented cells, and the green arrows indicate over-segmented cells. The red-dashed circles indicate missing segmented cells. The GFT method tended to under-segment cells, as it seldom segmented touching cells. The MSL method segmented nearly all the touching boundaries of the touching cells, but tended to over-segment the cells by segmenting one cell into multiple cells. The TWANG method tended to under-segment the cells and even eliminated most voxels of one cell, which is highlighted with the red-dashed circles in Figure 7. Our proposed method could segment the touching cells with little over-segmentation, although some cells may not have been accurately segmented. We have tried to quantitatively measure the segmentation accuracy; however, the cell boundaries in our datasets were too blurred, and the ground-truth annotations marked by different people differed greatly. Therefore, we eliminated the quantitative comparisons.

**Figure 8:**
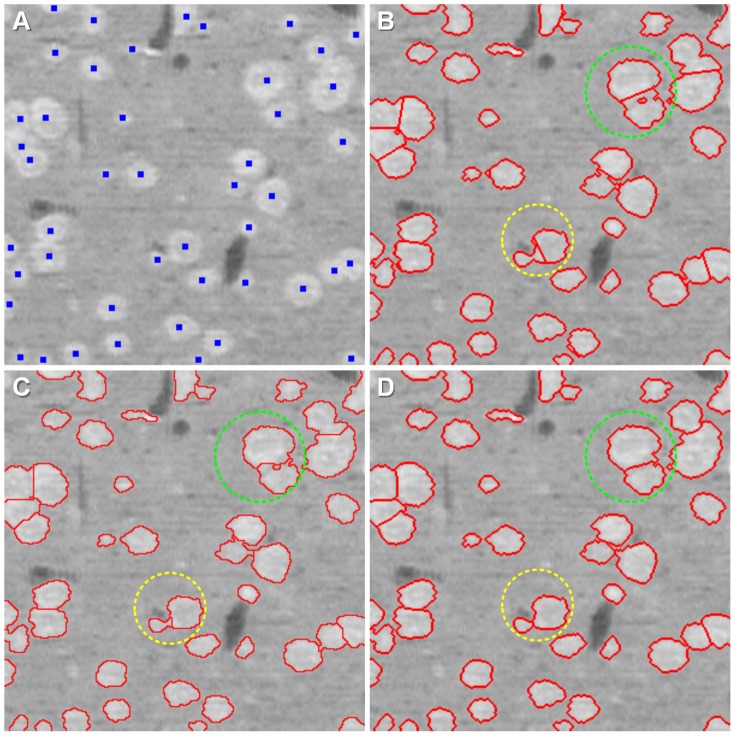
Comparison of the segmentation results using three different methods. The light green dashed circle indicates the holes in the binarization step, and the light yellow dashed circle indicates detached contours of two touching cells. (A) Seed point (blue points). The three segmentation methods are all based on the seed points. (B) The segmentation results of the k-mean algorithm, using seed points as the initial points. (C) The segmentation results of the marker-controlled watershed algorithm. (D) The segmentation results of the CC-random walker method.

Moreover, our seed point detection step could be combined with other segmentation algorithms, such as the k-mean and watershed algorithms, with the detected seed points as the initiation points or the initiation markers. We qualitatively compared the CC-random walker segmentation method with k-mean clustering (the seed point are the initiation points) and marker-controlled watershed segmentation (the seed points are the markers). The results of the three segmentation methods are shown in Figure 8, and the extracted cell contours are displayed in 2D sections in Figure 8B–D. The results demonstrated that the CC-random walker method was able to suppress the holes (light green dashed circles in Figure 8B–D) and obtain the exact contours of touching cells, but the k-mean clustering and watershed segmentation methods could not. Additionally, the contours of touching cells were biased to larger cells and deviated for smaller cells by the other two methods (light yellow dashed circles in Figure 8B–D).

## Discussion

The challenges of optical image segmentation behave as the heterogeneous brightness and irregular geometric characteristic. The brightness is often heterogeneous, however, in this study, the seed points were detected on binary images. Cells possess various geometric characteristics, and some of the cells have irregular shapes and touch significantly, which are prone to producing a poor detection result and under-segmentation. This study has addressed the problem of closely touching cells by introducing the CPCC method. The original 3D image stacks (the K1–K6 and C1–C20 stacks) as well as the detection and segmentation results are available for download from: http://bmp.hust.edu.cn/publication/pone2014_he.

We have compared the detection results of these state-of-the-art methods with our proposed CPCC method statistically. A Student's *t*-test is often used to test the average level difference of small sample sizes (where the sample number is less than 30). We used it to determine whether a significant difference existed between the detected results of our proposed and state-of-the-art methods. Three experiments (*t*-test comparisons) were performed: (1) our proposed CPCC method vs the GFT method; (2) our proposed CPCC method vs the MSL method; and (3) our proposed CPCC method vs the TWANG method. We first calculated the means and variances of the recall and precision metrics for the K1–K6 (Table 1) and C1–C20 (Table 2) stacks. And then the corresponding *p* values for the two metrics of CPCC vs GFT, CPCC vs MSL, and CPCC vs TWANG are presented in Table 3 with a confidence level of 0.05. We can conclude that both the recall and precision of our method were significantly higher than the GFT method. The recall of our method was significantly lower than the MSL method, but the precision of our method was significantly higher. For the K1–K6 stacks, no significant differences between our method and the TWANG method existed with respect to recall and precision. For the C1–C20 stacks, both the recall and precision of our method were significantly higher than the TWANG method.

**Table 3:** T-test results of our method vs GFT, our method vs MSL, our method vs TWANG under confidence level = 0.05.

Image Stack	T-test result	vs GFT [14]		vs MSL [3]		vs TWANG [38]	
		*R*	*P*	*R*	*P*	*R*	*P*
K1–6	S	+	+	−	+	no	no
	p-value	0.004	0.003	0.011	0.001	0.148	0.177
C1–20	S	+	+	−	+	+	+
	p-value	2.7×10^−13^	0.0001	0.008	0.0003	4.9×10^−09^	0.006

“*R*” means recall and “*P*” means precision. “S” means significance. “+” means significantly higher. “−” means significantly lower. “no” means no significant difference.

Next, the performance of our proposed method compared with the other state-of-the-art methods will be discussed. These state-of-the-art methods achieved excellent results in their applicable situations and all the discussions below were only limited to our datasets. As for the GFT method, the under-detection and under-segmentation were serious in our data. The main reason may be that the GFT method relies on a gradient, and since our data are heterogeneous, the gradient will not flow toward the cell centroid. Also for the GFT method, Al-Kofahi *et al* described that the rough chromatin texture may produce inaccurate flow values and/or directions in their experiments [3]. Qi *et al* assessed that the GFT method was not suitable for data containing large numbers of cells with extensive overlapping areas [11], which was verified in our datasets. The MSL method could detect and segment nearly all the cells, including the touching cells. However, false detection and over-segmentation were also significant, which were caused by the heterogeneous brightness in our datasets. The authors mentioned that over-segmentation usually occurs when a cell is chromatin–textured, and the shape is highly elongated. Both under and over segmentation occurred when the TWANG method was used with our data. One reason may be that this method trades, on a certain extent, accuracy for speed [38]. Also, as the authors previously described, the algorithm tends to clip the tips of elongated cells. Therefore, it is not suitable for the irregular cell shapes in our datasets. Our proposed cell detection and segmentation method is based on the binary image, which can avoid the influence of heterogeneous brightness. Moreover, we made use of the concave points to achieve seed points for touching cells, but some under-segmentation still occurred when the shape of touching cell was extremely irregular.

This study has yielded a CC-random walker method to segment cell contours. The method can overcome the holes in the image, even when the holes were very large. According to our analysis, the voxels in holes were generally surrounded by foreground voxels; thus, the probability of the voxels reaching the nearest foreground seed point was greater than the probability of the voxels reaching the nearest background seed point. Consequently, the voxel of the hole was labeled as foreground. In addition, by transforming the segmentation issue of the touching cells into an independent optimization problem, we obtained accurate detached contours for the touching cells.

There are three important parameters in the seed-point detection step discussed below. The first two parameters are *Eps* and *Minpts* in the Ckernel-DBSCAN algorithm. The value of *Minpts* is set to 4 in DBSCAN [32] (a little larger also works), and the value of *Eps* is determined by 4-dist graph or by some attempts. We chose *Minpts* = 5 and *Eps* = 3 after some attempts. When *Eps* and *Minpts* were set too large, the concave points of different touching-cell-pairs were falsely clustered into the same class, resulting in a reduction in the detection recall rate. When *Eps* and *Minpts* were set too small, the concave points of one touching-cell-pair were falsely clustered into different classes, causing the precision rate to be reduced. The third important parameter is *Rc*, which usually corresponds to the cell radius. We counted the volumes of 100 real isolated cells and approximated each isolated cell as a sphere to estimate the radius using the volume formula of a sphere. The average radius of the 100 cells was about *Rc* = 7 µm. If *Rc* was too small, the seed points were located too near to the cell contour, but if *Rc* was too large, the seed points were located too near to the junction of touching cells.

**Figure 9:**
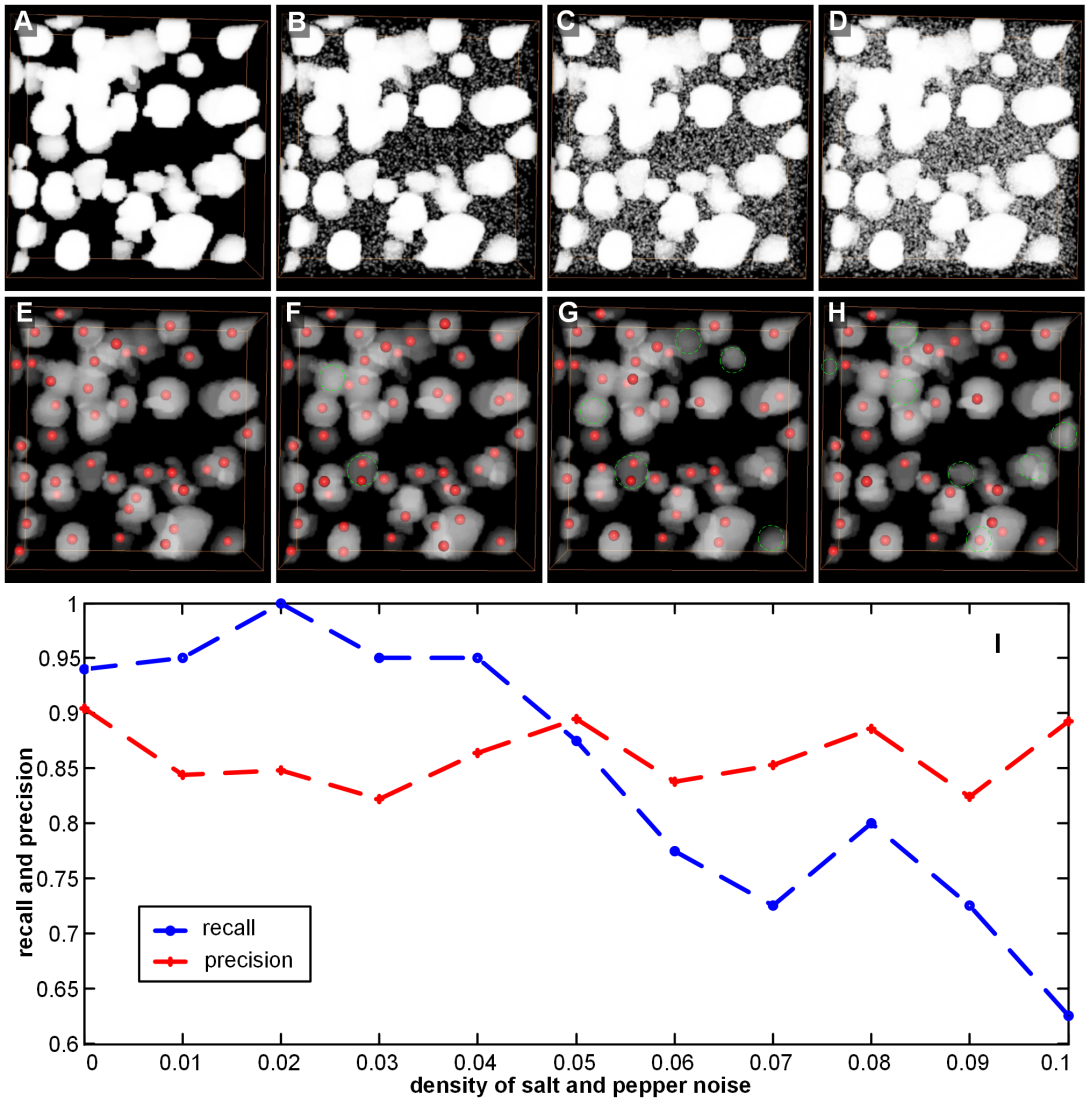
Noise influences on the precision and recall for the K1 stack. Salt and pepper noise is used here. The red point is the detected point, and the green dashed circle indicates a missing seed point caused by noise. (A–D) The volume-rendered (with the colormap's alpha values of 0.5) binary image stack with different levels of noise density, i.e., 0, 0.03, 0.06, and 0.09. (E–H) The seed point detection results with different levels of noise density, i.e., 0, 0.03, 0.06, and 0.09. For easy observation, the seed point is placed on the binary stack, pre-noise elimination, and the pre-noise elimination binary stack is volume-rendered with the color map's alpha values of 0.2. (I) Performance curves of the seed point detection estimation with varying noise densities.

To assess the general capabilities of our proposed method, we added noise into the stack and evaluated the seed point detection results. Because the seed point detection and segmentation steps were both performed on the binary image, we chose salt and pepper noise with noise density ranging from 0.00 to 0.10, i.e., {0, 0.01, 0.02, 0.03, 0.04, 0.05, 0.06, 0, 0.07, 0.08, 0.09, 0.10}, and the parameters of the original binary image stack and the noised binary image stack were the same. Finally, we computed the recall and precision values at each noise level. The seed point detection results of the K1 stack with some noise levels are shown in Figure 9A–E. We found that the detection results were influenced by noise to a certain extent. Some seed points were missing (see the green dashed circle in Figure 9F–H) when noise was added. Figure 9I shows the performance curves of the seed point detection estimation (Precision, Recall) for varying noise densities. The precision performance of the seed point detection varied with the added noise, but it never varied by more than 10%, and the recall performance was reduced with an increase in the noise density. In the Ckernel-DBSCAN algorithm, we needed to compute the density of concave points, and the addition of salt and pepper noise can result in false positives for concave points. Thus, the density of concave points was influenced, which may affect the cluster concave point results, for example, the high density of noise points caused concave points of different touching-cell-pairs to be clustered together, reducing the number of clusters and omitting the seed points of some touching cells (recall performance was reduced).

The detection and segmentation algorithms were implemented in MATLAB (Mathworks, Natick, MA). The demonstrated data were processed on a personal desktop workstation (Intel Xeon X5690/12 cores/3.46 GHz, 48GB). The computational complexity of each step in this method will now be discussed. The time and space complexity of the image enhancement, binarization, image filling and morphological filtering were all linear *O(n)*, where *n* was the number of image voxels. Connected-component analysis was implemented as a function in MATLAB, and the details of its computational complexity were unclear. The time and space complexity of concave point detection was *O(n+D*×*W^3^)*, where *W* was the size of the mask, M, and *D* was the number of contour points. The time and space complexity of the Ckernel-DBSCAN method was *O(D*×*k)*, where *k* was the number of each concave point's S_Eps_-neighbor. The process of achieving seed points was relative to the number of concave point clusters, and the complexity was *O(s)*, *s<<n*. The time complexity of the random walker segmentation was *O(N*×*f(n/N))*, where *N* was the number of connected components and *f(n/N)* was the complexity of solving *n/N* equations. The space complexity of the random walker segmentation was *O(N*×*(n/N)^4^)*. The most time consuming step during segmentation was due to solving sparse linear equations in the 3D image stack; for the K1 stack, the random walker segmentation time was 0.012 s.

There were also some problems with our seed point detection step. First, it was designed specifically for approximately spherical-shaped cells. Second, it was based on binary images, and thus, it is influenced by the binarization method. For isolated cells, when the contrast on the surface varies greatly, there may be many “false” concave points at the surface after binarization. These “false” concave points may cluster into classes using the CPCC point, and more than one seed may be detected (false positives). The potential solution is to combine cell volume or other information to detect isolated cells. Third, it may lead to more false negatives when cell touching is very complex. For example, suppose there are three touching cells, A, B, and C, with A and B touching each other, B and C touching each other, and A and C touching each other. The Ckernel-DBSCAN algorithm may cluster all the concave points into only one class, and then the number of detected seed points may be less than 3, which leads to under-segmentation in the segmentation step. This problem is very difficult to solve, and we should develop a more effective algorithm in the future. Finally, the lighting conditions of the imaging system also have some influence on the binariziation step, which further affects the detection of seed points and segmentation.

## Conclusions

We have proposed a complete pipeline for 3D detection and segmentation of touching cells. The algorithm used in this study had high recall and precision rates for seed point detection, even when the cells were closely touching in 3D space, because the neighbor points of the CPCC point had been used. Additionally, the CC-random walker algorithm was not sensitive to the holes, which is of significant benefit to the high accuracy of the cell contour segmentation.
